# Impact of Rh five-antigen–matched transfusion on alloimmunization and clinical outcomes in patients requiring repeated red blood cell transfusions: a prospective randomized controlled study

**DOI:** 10.3389/fmed.2026.1795865

**Published:** 2026-04-08

**Authors:** Xiaolu Xie, Ye Qin, Hanke Wang, Jun Zhang

**Affiliations:** Department of Transfusion Medicine, Affiliated Hospital of Yangzhou University, Yangzhou, Jiangsu, China

**Keywords:** randomized controlled study, red blood cell alloimmunization, repeated red blood cell transfusion, Rh antigen matching, transfusion management

## Abstract

**Background:**

Red blood cell (RBC) alloimmunization remains a significant challenge in patients requiring repeated transfusions, complicating transfusion procedures and increasing healthcare resource utilization. While extended Rh antigen matching has been proposed to mitigate alloimmunization, prospective data on its impact on transfusion-related outcomes and clinical management remain limited.

**Methods:**

In this prospective, randomized controlled study, patients requiring multiple RBC transfusions were randomly assigned to receive either conventional antigen matching or Rh five-antigen (D, C, c, E, e)–matched transfusions. The primary endpoint was the incidence of RBC alloantibody formation. Secondary endpoints included transfusion-related adverse reactions, as well as transfusion management outcomes such as RBC utilization, transfusion intervals, and length of hospital stay. Multivariable analyses were performed to adjust for potential confounders.

**Results:**

Patients in the Rh five-antigen–matched group demonstrated a significantly lower incidence of alloantibody formation and fewer transfusion-related adverse reactions compared to the conventional matching group. Additionally, Rh five-antigen matching was associated with reduced RBC utilization, longer transfusion intervals, and shorter hospital stays. After adjusting for relevant clinical variables, Rh five-antigen–matched transfusion remained independently associated with lower RBC utilization.

**Conclusion:**

In patients requiring repeated RBC transfusions, Rh five-antigen–matched transfusion was associated with a reduced risk of alloantibody formation and more favorable transfusion management outcomes. This strategy represents a viable transfusion management approach for patients at high immunological risk, and its long-term clinical value and cost-effectiveness should be further explored in multicenter studies.

## 1 Introduction

Repeated red blood cell (RBC) transfusion is an essential supportive therapy for patients with a wide range of chronic and severe diseases. However, transfusion-related alloimmunization remains a major challenge in clinical transfusion management. The formation of irregular alloantibodies not only complicates subsequent compatibility testing but may also lead to delayed hemolytic transfusion reactions, transfusion delays, and increased overall healthcare risk. Consequently, alloimmunization is widely recognized as a critical factor affecting patient safety and the quality of medical care.

The Rh blood group system is the second most clinically important RBC blood group system after the ABO system and comprises several highly immunogenic antigens, including D, C, c, E, and e (1). In routine transfusion practice, compatibility testing has traditionally focused on the RhD antigen, whereas the immunogenicity of non-D Rh antigens and their contribution to alloimmune responses have received comparatively less attention (2). Previous studies have shown that, in patients requiring chronic or repeated transfusions, reliance on ABO and RhD matching alone is often insufficient to prevent alloantibody formation, with a substantial proportion of antibodies directed against non-D Rh antigens.

In recent years, advances in red cell genotyping technologies have enabled more precise identification of multiple Rh antigens, thereby facilitating the implementation of extended antigen-matching strategies at the multi-antigen level. These developments have attracted increasing interest in both transfusion medicine and obstetric practice (3–5). Available evidence indicates that, in patients undergoing chronic or repeated transfusions, the use of molecular genotyping to identify RHD/RHCE variants and the application of extended Rh antigen matching—including C, c, E, and e—are significantly associated with a reduced risk of alloimmunization (6, 7). Among these antigens, non-D Rh antigens are generally characterized by relatively high immunogenicity and are particularly prone to inducing immune sensitization in the setting of repeated transfusion exposure.

Within the Rh system, the C and E antigens are considered to have strong sensitizing potential. Antibodies directed against these antigens not only increase the complexity of subsequent transfusion compatibility testing but may also precipitate hemolytic reactions and delayed hemolytic transfusion events (8). These immunological features are especially evident in pregnant populations, in whom Rh antibodies—particularly anti-D, anti-c, and anti-E—have been firmly established as major causes of hemolytic disease of the fetus and newborn (HDFN) (6).

The concept of precision transfusion emphasizes individualized transfusion strategies tailored to patient-specific characteristics, including RBC antigen profiles, prior alloantibody history, and transfusion exposure. By applying multi-antigen matching approaches, precision transfusion aims to reduce alloimmune responses and transfusion-related complications. Accumulating evidence suggests that, in patients at high immunological risk or those requiring repeated transfusions, ABO and RhD matching alone is often inadequate for preventing alloantibody formation, whereas non-D Rh antigens—such as C, c, E, and e—play a particularly critical role in alloimmunization (9). In international clinical practice, extended matching based on the five principal Rh antigens (D, C, c, E, and e) has been adopted in several patient populations requiring long-term or repeated transfusions. Multiple studies from the United States and Europe have consistently demonstrated that, compared with conventional matching strategies, non-D Rh antigen matching is significantly associated with lower rates of transfusion-related alloantibody formation and reduced transfusion management complexity (10–12).

Advances in molecular immunohematology have provided an essential technical foundation for the implementation of these strategies. Molecular techniques such as PCR with sequence-specific primers (PCR-SSP) and RHD/RHCE gene sequencing enable more accurate identification of complex or weakly expressed Rh antigen phenotypes, thereby overcoming the limitations of traditional serological typing in the context of repeated transfusions or antibody interference. These methods offer a reliable basis for precise multi-antigen Rh matching (1, 13). Nevertheless, despite continued progress in precision transfusion concepts and related technologies, widespread clinical implementation remains challenging. Limited availability of Rh multi-antigen–matched blood units, together with incomplete molecular blood group databases and supporting information management systems, continues to restrict the routine application of extended matching strategies (14).

Notably, most existing studies have primarily focused on the short-term effects of Rh five-antigen matching on alloantibody formation. Prospective evidence regarding its association with transfusion management–related outcomes—such as RBC utilization and transfusion intervals—as well as long-term antibody evolution in real-world clinical settings remains limited. Therefore, systematic evaluation of the overall clinical value of Rh five-antigen matching as a transfusion management intervention under randomized controlled conditions is warranted.

Against this background, the present study employed a prospective randomized controlled design to comprehensively evaluate the clinical application of Rh five-antigen (D, C, c, E, e)–matched transfusion in patients requiring repeated RBC transfusions. The study focused on alloantibody formation, transfusion-related adverse reactions, transfusion management–related outcomes, and long-term antibody dynamics, with the aim of providing more robust evidence to support precision transfusion strategies in patients at high immunological risk.

## 2 Materials and methods

### 2.1 Study population

This single-center, prospective, randomized controlled trial was conducted at the Affiliated Hospital of Yangzhou University from January 2022 to December 2024. Hospitalized patients requiring RBC transfusion therapy were screened for eligibility.

#### 2.1.1 Inclusion criteria

(1) age ≥18 years; (2) requirement for planned RBC transfusion due to chronic anemia (e.g., malignancy-associated anemia or bone marrow failure syndromes) or acute blood loss, with anticipated cumulative transfusion volume ≥4 units based on disease type and hemoglobin level (Hb < 80 g/L); (3) confirmed ABO and RhD blood group; (4) negative pre-transfusion irregular alloantibody screening; (5) written informed consent.

#### 2.1.2 Exclusion criteria

(1) history of severe transfusion-related adverse reactions; (2) autoimmune hemolytic anemia or inherited RBC membrane disorders; (3) pregnancy or lactation; (4) previously documented RBC alloantibodies.

Of 682 patients initially screened, 82 were excluded: 56 for failing inclusion criteria (e.g., anticipated transfusion volume <4 units, positive pre-transfusion antibody) and 26 for meeting exclusion criteria (pregnancy, lactation, prior RBC alloantibody, autoimmune hemolytic anemia, or history of severe transfusion reaction).

#### 2.1.3 Randomization

600 eligible patients were randomized 1:1 into the experimental group (Rh five-antigen–matched transfusion, *n* = 300) or control group (conventional ABO- and RhD-matched transfusion, *n* = 300). All patients received at least one RBC transfusion; 96.7% received ≥2 transfusions. Patients were further categorized by actual transfusion number: two (n = 402, 67%), three–four (n = 148, 24.7%), and ≥5 (n = 30, 5%).

#### 2.1.4 Ethics and trial registration

The study was approved by the Ethics Committee of the Affiliated Hospital of Yangzhou University (approval no. 2023-YKL12-DZX05) and conducted in accordance with the Declaration of Helsinki. This trial was not prospectively registered due to its single-center exploratory design; all eligibility criteria, interventions, and outcome assessments are fully described in the Methods to ensure transparency and reproducibility.

### 2.2 Randomization, blinding, and intervention

After obtaining written informed consent, eligible patients were randomly assigned in a 1:1 ratio to the experimental group (Rh five-antigen–matched transfusion, *n* = 300) or the control group (conventional ABO- and RhD-matched transfusion, *n* = 300). Randomization sequences were generated by an independent statistician using SPSS version 26.0 (a).

Allocation concealment was achieved using sequentially numbered opaque envelopes, managed solely by designated transfusion personnel who were not involved in patient recruitment, clinical care, or outcome assessment (b). These personnel were only responsible for revealing group assignment to prepare the corresponding blood units; all other clinical and outcome evaluation staff had no access to allocation information (c).

#### 2.2.1 Blinding

Complete blinding of treating clinicians was not feasible due to the nature of the transfusion intervention. Patients and outcome assessors were blinded to group assignment to minimize assessment bias. Laboratory personnel conducting antibody testing and independent evaluators were unaware of group allocation. Measures to maintain blinding included physical separation of outcome assessors from the transfusion service, labeling all samples with a study-specific ID independent of the randomization sequence, and anonymizing clinical adverse event reports before evaluation (d).

#### 2.2.2 Interventions

Control group patients received conventional ABO- and RhD-matched RBC transfusions, while experimental group patients received Rh five-antigen–matched transfusions (C, c, E, e, in addition to ABO and RhD matching).

#### 2.2.3 Primary endpoint definition and analysis

The primary endpoint was defined as the formation of clinically significant RBC alloantibodies within 6 months post-randomization. Only antibodies with potential clinical impact on transfusion safety (e.g., Rh or Kell system antibodies) were included. Low-titer, naturally occurring, or clinically irrelevant antibodies were excluded. All laboratory evaluations were independently confirmed and performed with personnel blinded to group allocation. Proportion comparisons between groups were performed using Chi-square tests, with multivariable logistic regression adjusting for relevant covariates, including age, sex, baseline hemoglobin, underlying diseases, and surgical interventions.

### 2.3 Sample size calculation

The sample size was estimated based on a pilot study conducted at the Affiliated Hospital of Yangzhou University from June 2021 to December 2021. The pilot included 60 patients requiring multiple RBC transfusions who met the inclusion criteria for the main study. The observed incidence of RBC alloantibody formation was 16.7% in the control group and 6.0% in the experimental group.

The sample size was calculated based on the primary endpoint of RBC alloantibody formation at 6 months. Proportions of alloimmunization in the control and experimental groups were compared using a Chi-square test. A Yates continuity correction was applied for conservative estimation.

The minimum sample size was calculated using the formula for comparing two independent proportions:


$$n=({\left(\mathrm{Z\alpha}/2+\mathrm{Z\beta}\right)}^{2}\times [\mathrm{p1}(1-\mathrm{p1})+\mathrm{p2}(1-\mathrm{p2})])/{\left(p1-\mathrm{p2}\right)}^{2}$$

Where p1 and p2 are the expected proportions in the control and experimental groups, Zα/2 corresponds to a two-sided type I error rate of 0.05, and Zβ corresponds to a power of 80%. Calculations were performed using PASS 15.0 software.

Based on anticipated proportions of 16.7% in the control group and 6.0% in the experimental group, the minimum required sample size was 270 per group. To account for potential loss to follow-up and maintain adequate power, the final target sample size was set at 300 per group (total 600 patients).

This approach ensures transparent and reproducible sample size determination for the randomized controlled trial.

### 2.4 Laboratory methods

#### 2.4.1 Blood group typing

All participants initially underwent routine serological blood group typing. ABO and RhD blood groups were determined using the microcolumn gel method. Rh system C, c, E, and e antigens were typed using Rh antigen detection cards with monoclonal antibodies (Changchun Baoxun Biotechnology Co., Ltd., China).

For samples with inconclusive serological typing results, RhD genotyping was further performed using a human RBC RhD genotyping kit based on the polymerase chain reaction with sequence-specific primers (PCR-SSP) method (Tianjin Xiupeng Biotechnology Development Co., Ltd., China).

Additional Rh molecular genotyping was performed under the following conditions: (1) weak D antigen expression (agglutination strength ≤2+) or mixed-field reactions; (2) inconsistent or ambiguous serological typing results for C/c/E/e antigens; and (3) a history of transfusion within 3 months prior to sampling or a prior pregnancy that could interfere with serological typing. Among the 600 enrolled patients, 82 cases (13.7%) underwent Rh molecular genotyping due to unclear serological results, weak antigen expression, or recent transfusion history. The remaining cases were typed and matched based solely on serological results.

In this study, Rh five-antigen matching was implemented as a predefined transfusion management intervention. Molecular genotyping served exclusively as a supplementary tool to serological testing in selected high-risk or diagnostically challenging cases and was not applied as a routine screening method.

#### 2.4.2 Alloantibody screening and identification

RBC alloantibody screening was performed using the microcolumn gel indirect antiglobulin test in combination with a 16-cell antibody identification panel (Immucor Gamma, United States). The screening panel covered clinically significant alloantibodies relevant to transfusion practice, including anti-C, anti-c, anti-E, anti-e, anti-K, anti-Fyᵃ, and anti-Jkᵃ.

Alloantibody testing was conducted at predefined time points: prior to transfusion (baseline) and at 1 week, 1 month, 3 months, 6 months, and 12 months after transfusion. All procedures were performed strictly in accordance with the manufacturers’ instructions. Specimens were centrifuged at 1200 × g for 10 min.

#### 2.4.3 Quality control and interpretation

Positive (anti-D serum) and negative (physiological saline) controls were included in each testing batch. All results were independently interpreted by two certified transfusion technologists. In cases of disagreement, a third senior technologist performed confirmatory review.

#### 2.4.4 Antibody titration and hemolysis monitoring

Samples positive for alloantibodies underwent antibody titration using either saline or antiglobulin methods with serial two-fold dilutions, starting at a dilution of 1:2. The endpoint was defined as the highest dilution at which agglutination was no longer observed.

Hemolysis-related criteria were defined as follows:

*Acute hemolytic reaction:* a decrease in hemoglobin >1 g/dL within 24 h after transfusion, accompanied by a haptoglobin level <25 mg/dL and a positive direct antiglobulin test (DAT).

*Delayed hemolytic reaction:* a decrease in hemoglobin >1 g/dL occurring 3–14 days after transfusion, together with an indirect bilirubin level >2 mg/dL and lactate dehydrogenase (LDH) > 250 U/L.

Antibody persistence was defined as detection of the same alloantibody in two consecutive follow-up tests separated by at least 1 month.

Acute hemolytic transfusion reactions were defined as immune- or non–immune-mediated hemolysis occurring during transfusion or within 24 h thereafter, typically accompanied by decreased haptoglobin levels and elevated LDH and bilirubin concentrations, with immune-mediated reactions usually demonstrating a positive DAT (1–3). Delayed hemolytic transfusion reactions generally occurred between 24 h and 28 days after transfusion and were characterized by an inadequate post-transfusion hemoglobin increment or a secondary decline, accompanied by laboratory evidence of hemolysis and newly detected RBC alloantibodies (4–6).

#### 2.4.5 Repeat testing and follow-up

A randomly selected 10% of samples underwent blinded repeat testing in a second laboratory to assess typing concordance. High concordance was observed, with a kappa coefficient of 0.98.

To evaluate long-term antibody dynamics, follow-up was extended to 24 months for all alloantibody-positive patients (n = 68), with additional testing at 18 and 24 months. Female patients of childbearing age (<50 years) and patients scheduled for elective surgery within 6 months underwent further follow-up extended to 36 months to document pregnancy outcomes or transfusion-related reactions following surgery.

Follow-up was conducted using a combination of automated reminders from the hospital information system and telephone contact, with a target loss-to-follow-up rate of less than 8%. If the loss-to-follow-up rate exceeded 10%, multiple imputation was applied to address missing data.

### 2.5 Transfusion procedure and handling of single-antigen mismatches (professionally polished)

Single-antigen mismatch was defined as donor antigen positive and recipient antigen negative. All RBC units were prioritized for ABO + RhD + Rh five-antigen (C, c, E, e) matching. When full antigen matching was not possible, mismatched units were recorded as single-antigen mismatches and transfused as clinically indicated. All such events were logged in the LIMS system to allow monitoring and inclusion in multivariable analyses.

Multivariable regression analyses adjusting for age, sex, baseline hemoglobin, underlying diseases, and surgical interventions were performed to assess the potential impact of mismatched transfusions on alloantibody formation. The observed reduction in alloantibody formation in the experimental group is consistent with the mechanism that reducing single-antigen mismatches limits exposure to novel antigens, thereby decreasing the probability of alloimmunization.

### 2.6 Blinded outcome assessment

The primary outcome (alloantibody formation) and secondary outcomes (transfusion-related adverse reactions) were assessed by an independent evaluation team under blinded conditions. Specifically, blood samples and clinical information from patients with suspected transfusion reactions were submitted to the transfusion service by the treating departments. After routine processing, samples designated for antibody testing were anonymized, retaining only the study identification number, and subsequently transferred to an independent assessment laboratory.

Laboratory personnel interpreted test results solely on the basis of sample identification numbers and assay findings. All conclusions were independently reviewed and confirmed by a senior investigator who was blinded to treatment allocation, thereby ensuring strict separation between outcome assessment and clinical intervention.

### 2.7 Randomization, blinding, and information isolation

This prospective randomized controlled study strictly adhered to principles of randomization, blinding, and information isolation to minimize potential bias.

#### 2.7.1 Randomization and allocation concealment

Randomization was performed using a computer-generated block randomization sequence (block size = 6) created by an independent statistician with SAS version 9.4. Randomization codes were sealed in opaque, sequentially numbered envelopes and securely stored by personnel not involved in the study. After confirmation of eligibility and acquisition of written informed consent, the study coordinator sequentially opened the envelopes to assign participants to the respective groups, thereby ensuring allocation concealment.

#### 2.7.2 Blinding

A single-blind design was adopted. Both patients and their treating physicians were unaware of group allocation. Designated transfusion service personnel responsible for blood allocation were aware of group assignment; however, their role was strictly limited to implementation of the matching protocol, without involvement in clinical management, data collection, or outcome assessment.

#### 2.7.3 Information isolation for outcome assessment

To ensure objectivity in outcome determination, a multilayered information isolation strategy was implemented: (1) outcome assessments were conducted by independent investigators who were fully blinded to group allocation and physically separated from the transfusion service; (2) all antibody screening and identification samples were labeled using a study-specific identification system independent of the randomization sequence; and (3) clinical adverse event reports were stripped of any non-essential information that could potentially reveal group allocation prior to evaluation.

Through these safeguards, clear separation was maintained among personnel responsible for randomization and blood allocation, clinical management, and outcome assessment, thereby minimizing the risks of measurement and information bias.

### 2.8 Statistical analysis

#### 2.8.1 Sample size estimation

Sample size estimation was based on pilot data (*n* = 60), in which the incidence of alloantibody formation was 16.7% in the control group and 6.0% in the experimental group. Assuming a two-sided significance level of *α* = 0.05 and a statistical power (1 − *β*) of 80%, the minimum required sample size was calculated as 586 patients using a two-sample proportion comparison formula. Allowing for an anticipated dropout or loss-to-follow-up rate of approximately 10%, the final target sample size was set at 600 patients.

#### 2.8.2 Data management and statistical software

All study data were entered, managed, and analyzed using SPSS version 26.0 (IBM Corp., Armonk, NY, United States). All statistical tests were two-sided, and a *p* value <0.05 was considered statistically significant unless otherwise specified.

#### 2.8.3 Descriptive statistics and between-group comparisons

Continuous variables were first assessed for distribution using the Shapiro–Wilk test. Normally distributed data were expressed as mean ± standard deviation (SD) and compared using independent-samples t tests. Non-normally distributed data were expressed as median (interquartile range [IQR]) and compared using the Mann–Whitney U test.

Categorical variables were presented as counts and percentages [n (%)]. Between-group comparisons were performed using the Pearson χ^2^ test or Fisher’s exact test, as appropriate.

#### 2.8.4 Multivariable analysis of alloantibody formation

Binary logistic regression was applied to identify independent factors associated with alloantibody formation. Variables entered into the model included Rh matching strategy (precision matching vs. conventional matching), number of transfusions (continuous variable), age, sex, and the presence of underlying malignancy (yes/no).

Model calibration was assessed using the Hosmer–Lemeshow goodness-of-fit test (χ^2^ = 6.82, *p* = 0.558). Multicollinearity was evaluated using variance inflation factors (VIFs), with all VIF values <2.0, indicating the absence of significant multicollinearity.

Sensitivity analyses were conducted to assess model robustness. First, continuous variables with non-normal distributions (e.g., RBC utilization) were log-transformed and re-entered into the model, resulting in changes in key regression coefficients of <5%. Second, extreme observations involving >10 transfusions were excluded, and the regression model was re-estimated, with fluctuations in odds ratios (ORs) of <8%. These analyses supported the stability of the model estimates.

#### 2.8.5 Subgroup analyses and interaction testing

Exploratory subgroup analyses were pre-specified for age (<65 vs. ≥ 65 years), sex, baseline hemoglobin, and specific comorbidities such as chronic kidney disease or malignancy. The study was not powered for confirmatory inference within these subgroups, as the sample size was calculated for the primary endpoint of RBC alloantibody formation at 6 months.

Confirmatory analyses refer to the primary endpoint comparisons, whereas exploratory analyses refer to these pre-specified subgroup comparisons. Observed trends in the subgroups were interpreted with caution, and no causal conclusions were drawn. Adjusted effect estimates, where applicable, were obtained using multivariable regression analysis controlling for relevant covariates.

Predefined subgroup analyses were performed to evaluate potential effect heterogeneity. Stratification was conducted according to underlying disease status (malignancy vs. non-malignancy) and transfusion burden (≤4 vs. > 4 transfusions). Within each subgroup, the incidence of alloantibody formation and relative risks (RRs) were calculated.

For categorical outcomes, pooled RRs were estimated using the Cochran–Mantel–Haenszel method. For continuous outcomes, subgroup effects were evaluated using meta-regression techniques.

Interaction terms (precision matching × malignancy status and precision matching × transfusion burden) were incorporated into the multivariable logistic regression model to assess effect modification, using the following specification:


$$\begin{array}{l} \text{logit}(P)=\beta 0+\beta 1X1+\beta 2X2+\beta 3(X1\times X2)\backslash \\ \text{text}\{\text{logit}\}(P)=\backslash \text{beta}\_0+\backslash \\ \text{beta}\_1\;X\_1+\backslash \text{beta}\_2\;X\_2+\\ \backslash \text{beta}\_3\;(X\_1\backslash \text{times}\;X\_2)\text{logit}(P)\\ =\beta 0+\beta 1X1+\beta 2X2+\beta 3(X1\times \mathrm{X2}) \end{array}$$

Where X1X_1X1 denotes the Rh precision matching strategy and X2X_2X2 represents the stratification variable. Interaction effects were evaluated using likelihood ratio tests. Given the exploratory nature of these analyses, a threshold of *p* < 0.10 was applied to define statistical significance. Multiple comparisons were adjusted using the Bonferroni method.

For subgroups with very small sample sizes (e.g., *n* < 10), only descriptive statistics (means, standard deviations, or proportions) were calculated. No inferential statistics were performed, and these findings are reported purely for observational purposes.

#### 2.8.6 Handling of missing data

Missing data for the primary and secondary endpoints were carefully evaluated. For the intention-to-treat analysis, missing primary endpoint data were handled conservatively: participants with incomplete follow-up were treated as not having experienced the event. Sensitivity analyses were performed using multiple imputation to assess the robustness of the findings. Missing baseline covariates were imputed using predictive mean matching. All analyses were conducted using R version 4.2.1 and SPSS 26.0.

All statistical analyses for the primary endpoint, including proportion comparisons and multivariable logistic regression, were reviewed by an independent statistician. Sample size calculations are transparently reported in the Methods (2.8.1), with a minimum of 270 patients per group increased to 300 per group to account for potential loss to follow-up.

Subgroup analyses were carefully designed. Pre-specified subgroups were analyzed as planned, whereas other secondary or complex subgroups not pre-specified were explicitly labeled as exploratory. These analyses are interpreted cautiously, and no causal inference is made. Corresponding tables indicate exploratory analyses and adjusted effect estimates where applicable.

### 2.9 Long-term follow-up analysis of alloantibody outcomes

Among patients who developed alloantibodies, long-term follow-up analyses were conducted to evaluate antibody persistence, seroreversion, and recurrence. The duration of antibody persistence was estimated using the Kaplan–Meier method, and persistence curves were generated. Between-group differences were assessed using the log-rank test.

Recurrence was defined as reappearance of the same antibody specificity after prior seroreversion. Because some patients experienced multiple recurrence events during follow-up, the Andersen–Gill extension of the Cox proportional hazards model was applied to estimate hazard ratios (HRs) for recurrence. To account for the potential impact of death or loss to follow-up, these events were treated as competing risks, and Fine–Gray subdistribution hazard models were used to evaluate independent factors associated with antibody persistence.

To assess the dose–response relationship between transfusion burden and alloantibody formation, the Cochran–Armitage trend test was applied. A significant increasing trend in alloantibody incidence was observed with increasing numbers of transfusions (P for trend <0.001), indicating a cumulative sensitization effect.

Multivariable binary logistic regression analysis was further performed to identify independent factors associated with alloantibody formation, incorporating Rh matching strategy (precision matching vs. conventional matching), number of transfusions, underlying disease type (malignancy vs. non-malignancy), age, and sex. The analysis demonstrated that Rh precision matching was significantly associated with a reduced risk of alloantibody formation (OR = 0.231, *p* < 0.001).

Variance inflation factors for all covariates were below 2.0, indicating no significant multicollinearity and supporting the reliability of the regression estimates.

## 3 Results

### 3.1 Study flow diagram of Rh precision transfusion

**Figure 1:**
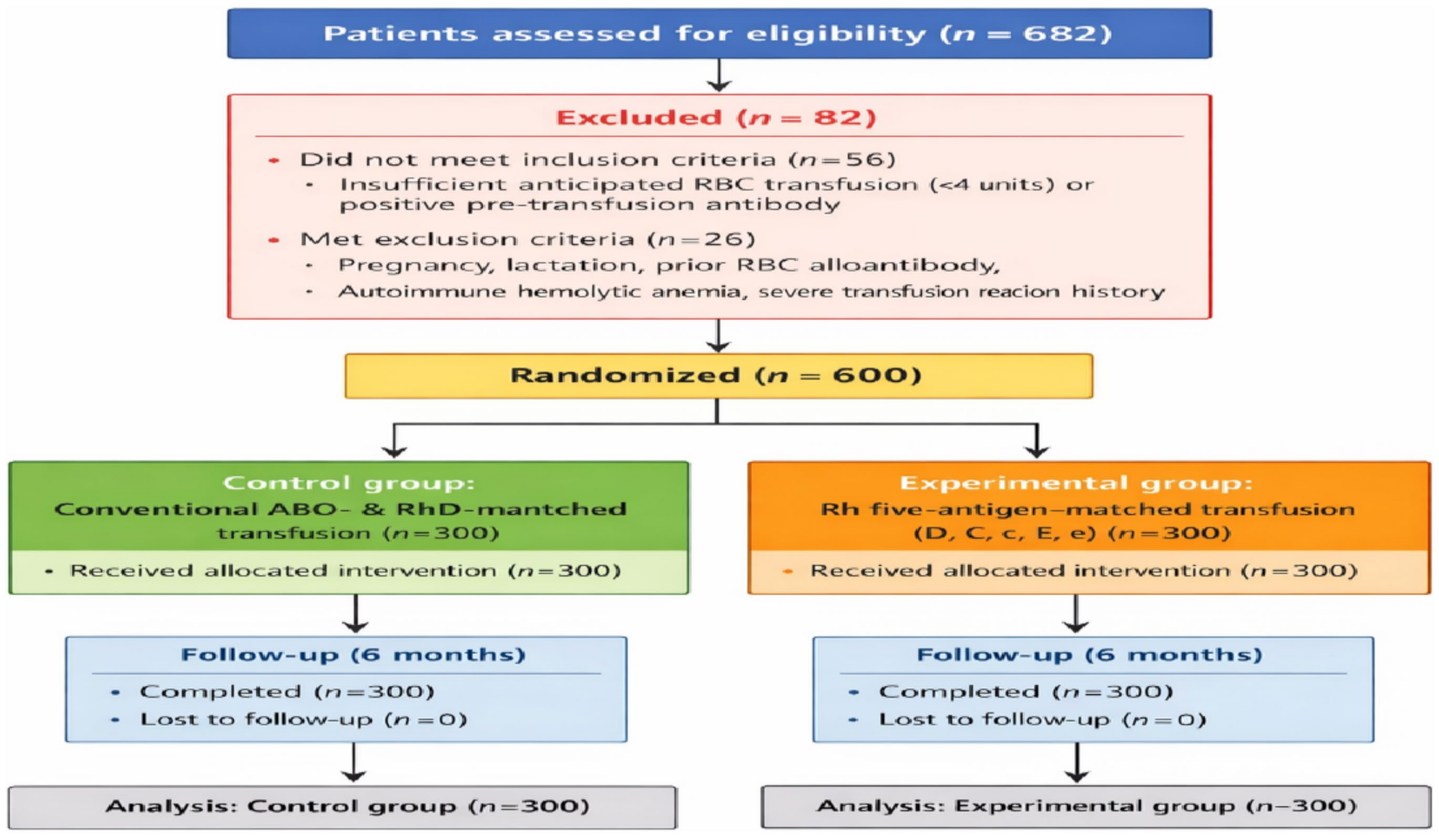
Flowchart of the Rh-matched transfusion study.

The study flow diagram of Rh precision transfusion is shown in Figure 1.

### 3.2 Comparison of baseline clinical characteristics and underlying diseases

A total of 600 patients were enrolled and randomly assigned to the experimental group (Rh five-antigen matched, *n* = 300) or the control group (conventional ABO and RhD matched, *n* = 300). In the control group, 162 patients (54.0%) were male and 138 (46.0%) were female; in the experimental group, 158 patients (52.7%) were male and 142 (47.3%) were female. There was no significant difference in sex distribution between groups (χ^2^ = 0.06, *p* = 0.806).

The mean age was 52.3 ± 13.6 years in the control group and 51.8 ± 13.1 years in the experimental group, with no statistically significant difference (t = 0.46, *p* = 0.647).

Malignancies were further classified into hematological malignancies and solid organ malignancies: in the control group, 54 (18.0%) and 39 (13.0%) patients, respectively; in the experimental group, 52 (17.3%) and 45 (15.0%) patients, respectively. The distribution of underlying diseases was balanced, and all between-group comparisons were non-significant (*p* > 0.05), indicating good randomization balance randomization. Detailed baseline characteristics are summarized in Table 1.

**Table 1:** Clinical characteristics of patients in the two groups.

Variable	Control group (*n* = 300)	Experimental group (*n* = 300)	Statistic	*P* value
Sex				
Male, *n* (%)	162 (54.0%)	158 (52.7%)	0.06	0.806
Female, *n* (%)	138 (46.0%)	142 (47.3%)	—	—
Age (years, mean ± SD)	52.3 ± 13.6	51.8 ± 13.1	t = 0.46	0.647
Underlying diseases, *n* (%)				
Malignancy	93 (31.0%)	97 (32.3%)	0.07	0.792
Hematological malignancies	54 (18.0%)	52 (17.3%)	—	—
Solid organ malignancies	39 (13.0%)	45 (15.0%)	—	—
Hematologic disorders	54 (18.0%)	52 (17.3%)	0.01	0.915
Renal insufficiency	41 (13.7%)	39 (13.0%)	0.01	0.904
Chronic liver disease	26 (8.7%)	28 (9.3%)	0.02	0.887
Cardiovascular disease	43 (14.3%)	40 (13.3%)	0.06	0.813
Infectious disease	43 (14.3%)	44 (14.7%)	0.00	1.000

Malignancy is further classified into hematological malignancies and solid organ malignancies. Total numbers are broken down by sex; percentages are based on each group (*n* = 300).

### 3.3 Alloantibody formation at 6 months (primary) and at 12 months/long-term follow-up (secondary/exploratory)

The incidence of RBC alloantibody formation was substantially lower in the experimental group receiving Rh five-antigen–matched transfusions compared with the control group, and this trend was consistent across follow-up time points.

At the 6-month primary endpoint, the experimental group showed a marked reduction in alloimmunization relative to controls. Follow-up assessments at 12 months and during long-term monitoring (up to 24 months) served as secondary or exploratory endpoints, confirming the durability of this protective effect.

These findings highlight the benefit of Rh five-antigen–matched transfusions in reducing alloantibody formation. Detailed numeric results and the follow-up schedule are presented in Table 2, and the visual flow of follow-up time points and endpoint classification is illustrated in Figure 1.

**Table 2:** Comparison of alloantibody formation between the two groups.

Antibody specificity	Control group (*n* = 300)	Experimental group (*n* = 300)	*P* value
Anti-C	20 (6.7%)	5 (1.7%)	0.003
Anti-E	12 (4.0%)	4 (1.3%)	0.041
Anti-c	10 (3.3%)	2 (0.7%)	0.016
Other antibodies	8 (2.7%)	7 (2.3%)	0.792
Total	50 (16.7%)	18 (6.0%)	< 0.001

Other antibodies include anti-D, anti-Fyᵃ, anti-Jkᵃ, and other low-frequency antibodies. Percentages are based on *n* = 300 per group, total *n* = 600.

### 3.4 Distribution of actual transfusion frequency and its association with alloantibody formation

The distribution of actual transfusion frequency and its association with alloantibody formation in the two groups are summarized in Table 3.

**Table 3:** Distribution of transfusion frequency and alloantibody formation in the two groups.

Number of transfusions	Control group (*n* = 300)	Experimental group (*n* = 300)	Total (*n* = 600)	Alloantibody formation rate (%), overall
1	10 (3.3%)	10 (3.3%)	20 (3.3%)	0.0% (0/20)
2	195 (65.0%)	207 (69.0%)	402 (67.0%)	7.0% (28/402)
3–4	80 (26.7%)	68 (22.7%)	148 (24.7%)	18.2% (27/148)
≥5	15 (5.0%)	15 (5.0%)	30 (5.0%)	43.3% (13/30)
Statistical test	χ^2^ = 1.32 (*p* = 0.724)	—	—	Trend χ^2^ = 58.1 (P < 0.001)

The comparison of alloantibody formation rates across transfusion frequency categories was performed using the Cochran–Armitage trend test.

### 3.5 Incidence of transfusion-related adverse reactions

The types and severity of transfusion-related adverse reactions were compared between the two groups. The overall incidence of transfusion-related adverse reactions was significantly lower in the experimental group than in the control group (3.3% vs. 9.0%, χ^2^ = 7.37, *p* = 0.007).

With respect to reaction severity, grade I reactions were the most common in the control group, including febrile non-hemolytic transfusion reactions and mild skin rash/pruritus, accounting for 4.0 and 3.0% of cases, respectively. The corresponding proportions in the experimental group were lower (1.7 and 1.3%, respectively), although these differences did not reach statistical significance (*p* = 0.140 and *p* = 0.262, respectively).

Grade II reactions, including delayed hemolytic transfusion reactions and mild-to-moderate allergic reactions, occurred in 1.7 and 0.3% of patients in the control group, respectively, compared with 0.3 and 0% in the experimental group. No statistically significant differences were observed between groups for these reaction types. No grade III severe adverse reactions, such as acute hemolysis or severe allergic reactions, were observed in either group.

Detailed distributions of transfusion-related adverse reactions by type and severity are presented in Table 4.

**Table 4:** Types and severity of transfusion-related adverse reactions in the two groups.

Reaction grade	Adverse reaction type	Control group (*n* = 300)	Experimental group (*n* = 300)	Statistical test and statistic	*P* value
Grade I	Febrile non-hemolytic transfusion reaction	12 (4.0%)	5 (1.7%)	χ^2^ = 2.18	0.140
	Mild rash/pruritus	9 (3.0%)	4 (1.3%)	χ^2^ = 1.26	0.262
Grade II	Delayed hemolytic transfusion reaction	5 (1.7%)	1 (0.3%)	Fisher’s exact test	0.218
	Moderate allergic reaction	1 (0.3%)	0 (0.0%)	Fisher’s exact test	1.000
Grade III	Acute hemolytic transfusion reaction	0 (0.0%)	0 (0.0%)	—	—
	Severe allergic reaction	0 (0.0%)	0 (0.0%)	—	—
Total		27 (9.0%)	10 (3.3%)	χ^2^ = 7.37	0.007

### 3.6 Comparison of red blood cell utilization and transfusion intervals

To assess the potential impact of the Rh five-antigen matching strategy on blood resource utilization and transfusion scheduling, RBC utilization and transfusion intervals were analyzed using defined operational measures. RBC utilization was defined as the percentage of actual RBC units transfused relative to the expected requirement during hospitalization. Transfusion interval was defined as the number of days between consecutive RBC transfusions.

**Figure 2:**
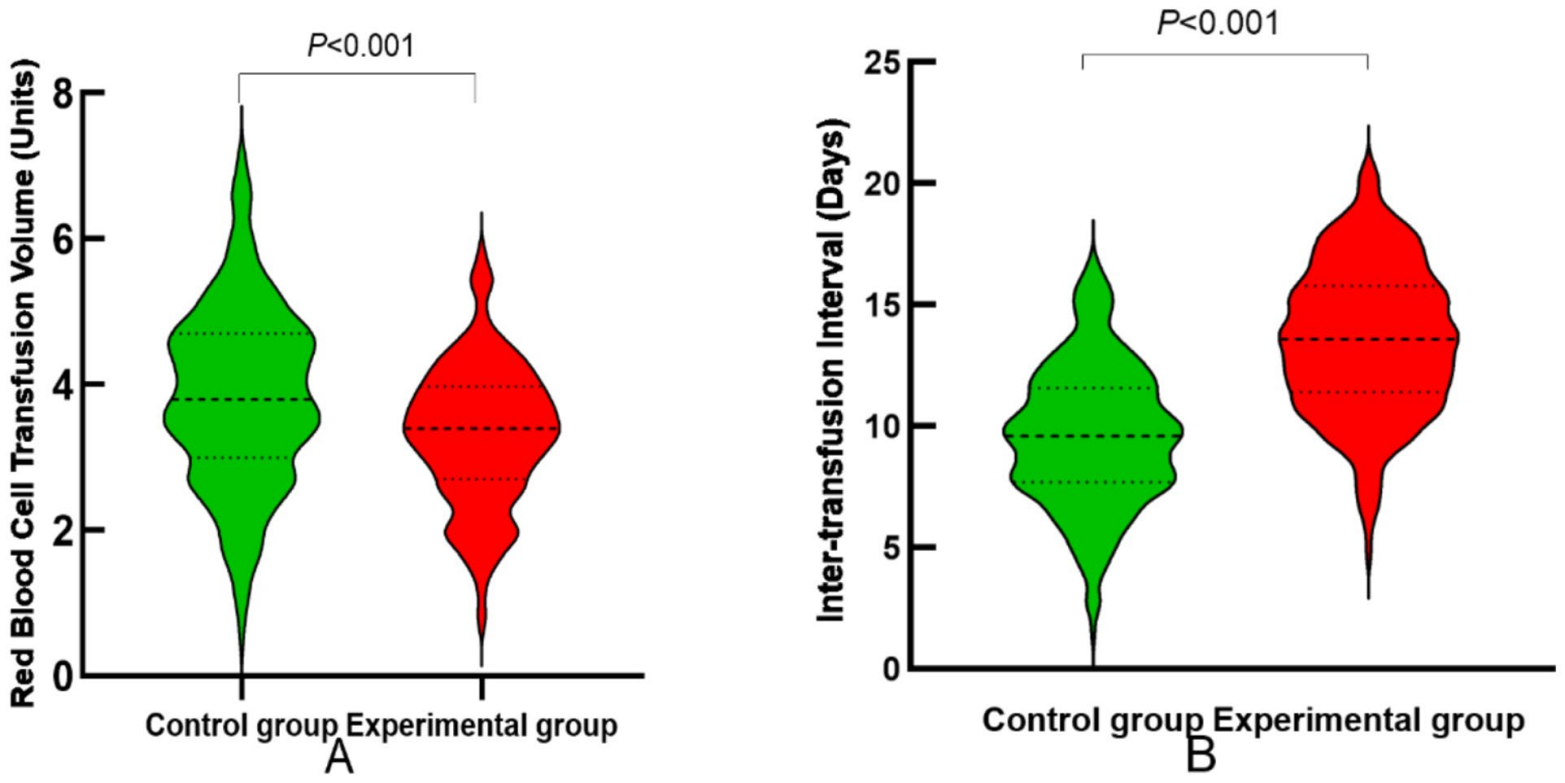
Comparison of red blood cell utilization and transfusion intervals. **(A)** Red blood cell utilization; **(B)** transfusion interval duration.

Multivariable regression analyses adjusting for age, sex, baseline hemoglobin, underlying diseases, and surgical interventions were performed to account for potential confounding factors. The experimental group consistently demonstrated lower RBC utilization and longer transfusion intervals compared with the control group. These trends are illustrated in Figure 2, and adjusted effect estimates are reported in Table 5.

**Table 5:** Red blood cell utilization, transfusion interval, and length of hospital stay (including single-antigen mismatch analysis).

Outcome	Control group (*n* = 300)	Experimental group (*n* = 300)	Adjusted effect (95% CI)	*P* value
RBC utilization (units)	3.9 ± 1.2	3.3 ± 0.9	−0.60 (−0.75, −0.45)	<0.001
Transfusion interval (days)	9.6 ± 2.9	13.7 ± 3.2	+4.1 (3.7, 4.5)	<0.001
Length of hospital stay (days)	12.5 ± 3.4	11.2 ± 3.1	−1.3 (−1.8, −0.8)	<0.001
Single-antigen mismatch events (%)	5 (1.7%)	2 (0.7%)	—	—

(1) RBC utilization = actual RBC units transfused ÷ expected RBC units × 100%. (2) Transfusion interval = number of days between consecutive RBC transfusions. (3) Length of hospital stay = total days from admission to discharge. (4) Single-antigen mismatch events recorded when donor antigen was positive and recipient antigen negative. (5) Adjusted effect estimates obtained using multivariable regression analysis, adjusting for age, sex, baseline hemoglobin, underlying diseases, and surgical interventions. (6) These results are associations, not causal effects.

These results indicate an association between Rh five-antigen–matched transfusion and reduced RBC utilization and prolonged transfusion intervals. However, these parameters reflect composite transfusion management outcomes influenced by multiple clinical and organizational factors. Therefore, the observed associations should be interpreted as correlative rather than causal, and should not be directly inferred as evidence of improved overall patient prognosis.

### 3.7 Comparison of length of hospital stay

The mean length of hospital stay was 14.2 ± 3.5 days in the experimental group and 15.4 ± 4.0 days in the control group. The between-group difference was statistically significant (t = 3.23, *p* = 0.001).

**Figure 3:**
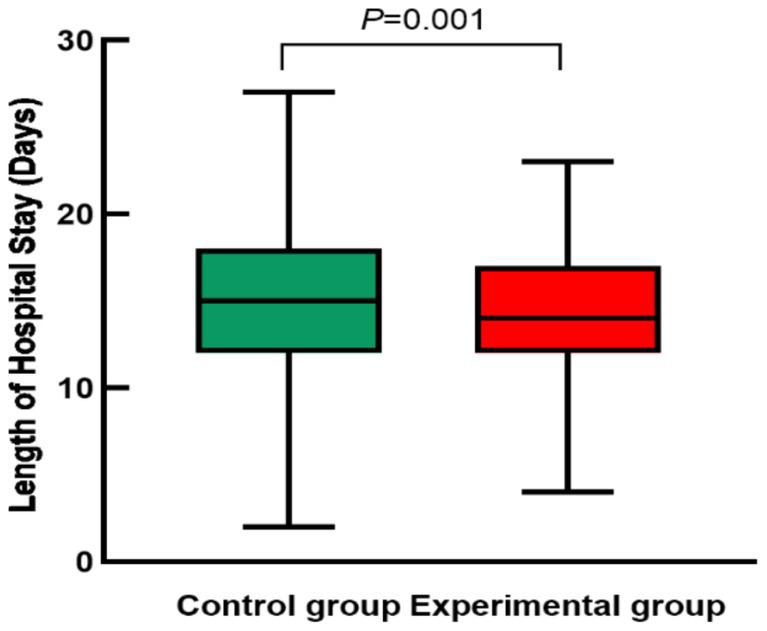
Comparison of hospital length of stay between the two groups.

It should be noted that length of hospital stay is influenced by multiple factors, including the severity of underlying diseases, treatment strategies, and clinical decision-making. Therefore, the present findings primarily reflect a statistical association between the Rh matching strategy and length of hospitalization, rather than a direct causal effect. These results are illustrated in Figure 3.

### 3.8 Impact of alloantibody formation on transfusion efficiency

After adjustment for potential confounders, Rh five-antigen–matched transfusion was independently associated with lower red blood cell (RBC) utilization (*β* = −0.61, 95% CI: −0.90 to −0.32, *p* < 0.001). Conversely, after multivariable adjustment, alloantibody formation was independently associated with increased RBC utilization (β = 0.82, 95% CI: 0.54–1.10, *p* < 0.001).

Together, these findings indicate that both the transfusion matching strategy and alloantibody status are independently associated with transfusion efficiency–related outcomes.

### 3.9 Multivariable logistic regression analysis of alloantibody formation

A multivariable logistic regression model was constructed with alloantibody formation as the dependent variable (0 = no alloantibody formation; 1 = alloantibody formation). Independent variables included Rh precision matching (yes vs. no), number of transfusions, underlying disease type (malignancy vs. non-malignancy), age, and sex. The results of the regression analysis are summarized in Table 6.

**Table 6:** Multivariable logistic regression analysis of factors associated with alloantibody formation.

Variable	β coefficient	OR (95% CI)	*P* value
Rh precision matching (yes vs. no)	−1.465	0.231 (0.126–0.421)	< 0.001
Number of transfusions (per additional transfusion)	0.266	1.305 (1.149–1.482)	< 0.001
Underlying malignancy (yes vs. no)	0.638	1.893 (1.058–3.385)	0.031
Age (years)	0.007	1.007 (0.986–1.029)	0.435
Sex (male vs. female)	0.152	1.164 (0.684–1.980)	0.572

#### 3.9.1 Key results

Rh precision matching emerged as an independent protective factor against alloantibody formation (OR = 0.231, *p* < 0.001). In contrast, an increasing number of transfusions and the presence of underlying malignancy were independently associated with a higher risk of alloantibody development. No significant associations were observed between alloantibody formation and patient age or sex.

### 3.10 Impact of alloantibody status on red blood cell utilization and transfusion intervals (subgroup analysis)

To further evaluate the potential influence of alloantibody status on transfusion efficiency, a subgroup analysis was conducted by stratifying the study population according to alloantibody formation: patients with alloantibodies (n = 68) and those without alloantibodies (*n* = 532).

Red blood cell (RBC) utilization was significantly higher among patients who developed alloantibodies compared with those who did not (4.15 ± 0.99 units vs. 3.30 ± 0.88 units, respectively; t = 7.37, *p* < 0.001).

Likewise, the mean transfusion interval was significantly shorter in the alloantibody-positive group than in the alloantibody-negative group (9.38 ± 2.67 days vs. 13.78 ± 2.98 days, respectively; t = −11.58, *p* < 0.001).

Taken together, these findings indicate that alloantibody formation is independently associated with increased RBC requirements and shortened transfusion intervals, underscoring alloantibody status as an important determinant of transfusion efficiency–related outcomes.

### 3.11 Subgroup analyses and interaction testing

#### 3.11.1 Subgroup analyses

**Figure 4:**
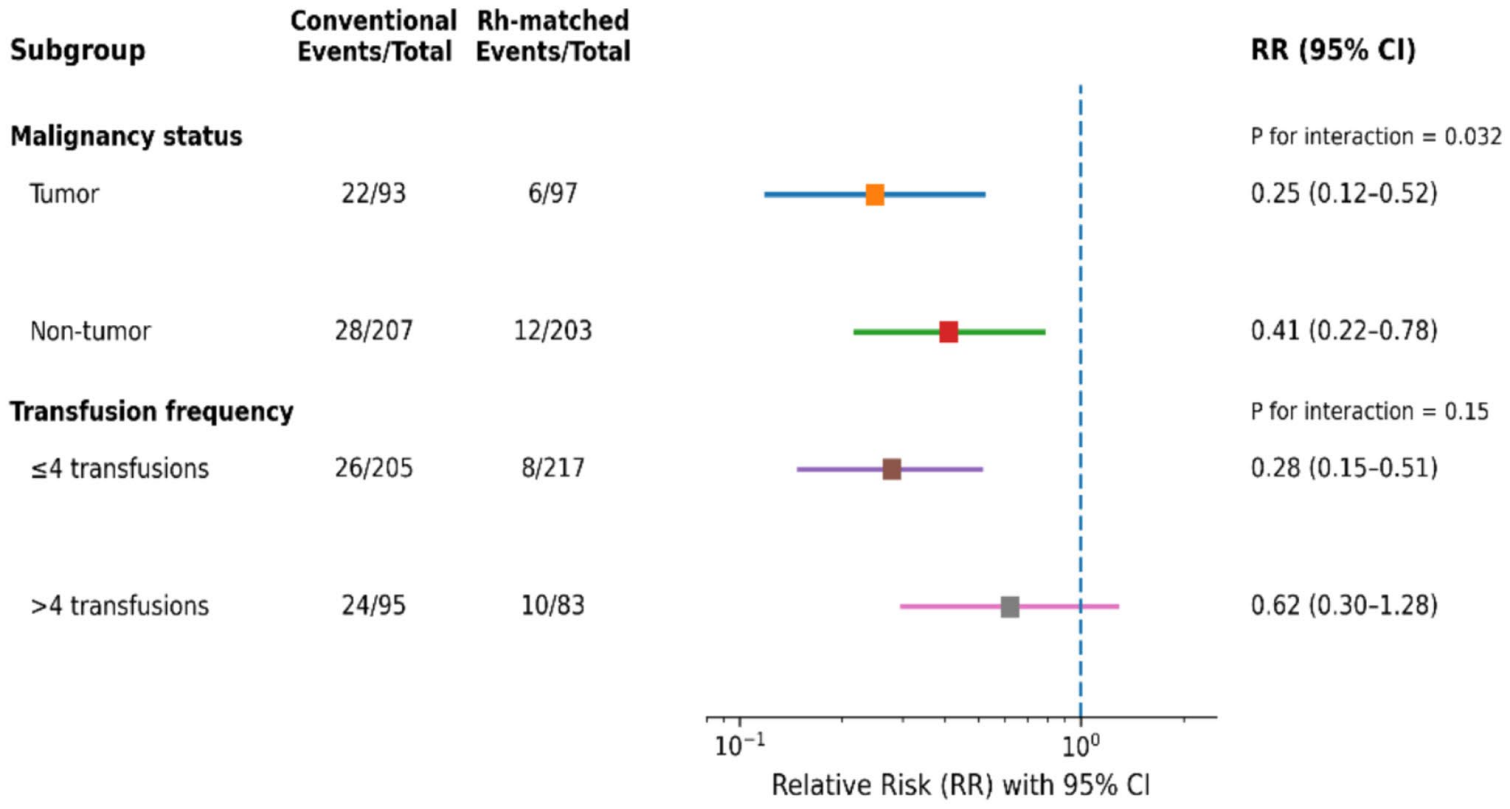
Subgroup analyses assessing the association between Rh five-antigen–matched transfusion and the risk of red blood cell alloantibody formation. Relative risks (RRs) with 95% confidence intervals (CIs) are shown for each subgroup on a logarithmic scale. Subgroups were defined according to underlying malignancy status (tumor vs. non-tumor) and transfusion frequency (≤4 vs. >4 transfusions). Numbers of events and total patients in the conventional and Rh-matched groups are provided for each subgroup. The vertical dashed line indicates a relative risk of 1.0, representing no difference between transfusion strategies. Interaction testing demonstrated significant effect modification by malignancy status (P for interaction = 0.032), whereas no significant interaction was observed for transfusion frequency (P for interaction = 0.15).

Subgroup analyses were performed using relative risks (RRs) with corresponding 95% confidence intervals (CIs), as illustrated in Figure 4. The protective association of Rh precision matching against alloantibody formation was more pronounced in patients with underlying malignancies (RR = 0.25, 95% CI: 0.12–0.52) than in those without malignancy (RR = 0.41, 95% CI: 0.22–0.78).

Among patients receiving more than four transfusions, Rh precision matching remained associated with a reduced risk of alloantibody formation (RR = 0.62); however, the magnitude of this association was attenuated compared with patients receiving four or fewer transfusions (RR = 0.28).

Exploratory subgroup analyses were performed for age, sex, baseline hemoglobin, and selected comorbidities. Consistent trends with the primary outcome were observed, but the study was not powered for confirmatory inference within these subgroups. Therefore, the findings should be interpreted as observational associations, and no causal conclusions can be drawn.

Both pre-specified and exploratory subgroup analyses were performed. Pre-specified subgroups showed trends consistent with the primary endpoint, while exploratory subgroups are presented for hypothesis generation only. The study was not powered for confirmatory inference within any subgroup, and no causal conclusions can be drawn.

#### 3.11.2 Interaction testing

Formal interaction testing demonstrated a statistically significant interaction between Rh precision matching and underlying malignancy status (P for interaction = 0.032), indicating that patients with malignancies derived greater relative benefit from precision matching. In contrast, no statistically significant interaction was observed between Rh precision matching and transfusion burden (P for interaction = 0.15).

#### 3.11.3 Visualization of subgroup effects

The subgroup-specific associations are summarized graphically in a forest plot (Figure 4).

### 3.12 Associations of alloantibody titer, persistence, and clinical outcomes

The associations between alloantibody titer, antibody persistence, and clinical outcomes were further evaluated. The results are summarized in Tables 7, 8.

**Table 7:** Associations of alloantibody titer and persistence with hemolytic outcomes.

Parameter	Alloantibody-positive patients (*n* = 68)	Patients with hemolytic reactions (*n* = 6)	Statistic	*P* value
Peak antibody titer (median, IQR)				
Anti-C	8 (4–32)	32 (16–64)	Z = 2.71	0.007
Anti-E	4 (2–16)	16 (8–32)	Z = 2.18	0.029
Antibody persistence				
Transient antibodies (< 3 months)	39 (57.4%)	0 (0%)	—	—
Persistent antibodies (≥ 3 months)	29 (42.6%)	6 (100%)	RR = ∞	< 0.001
Association between titer and hemolysis severity				
Maximum hemoglobin decline (g/dL)	—	2.8 ± 0.6	r = 0.89*	< 0.001
Peak LDH (U/L)	—	586 ± 142	r = 0.78*	0.001

*Correlation analysis performed using Spearman’s rank correlation test.

**Table 8:** Associations between alloantibody status and clinical outcomes.

Alloantibody status	Reoperation cases (*n*)	Transfusion reaction rate	Pregnancy cases (*n*)	HDFN incidence
Persistently negative	85	2.1%	25	0%
Seroconversion or reappearance	27	18.5%	2	100%

Due to the limited sample size in analyses involving pregnancy outcomes and hemolytic disease of the fetus and newborn (HDFN), these results are presented for descriptive purposes only. No inferential statistical analyses were performed, and the findings should be interpreted with caution. Confirmation in larger cohorts is warranted.

### 3.13 Alloantibody resolution and recurrence analysis

Among all alloantibody-positive patients (*n* = 68), the rate of antibody resolution was significantly higher in the experimental group than in the control group. At 12 months of follow-up, the cumulative seroreversion rate was 83.3% (15/18) in the experimental group, which was markedly higher than that observed in the control group (36.0%, 18/50; *p* < 0.001). By 24 months, this difference further widened (96.3% vs. 62.0%, log-rank *p* < 0.001).

Kaplan–Meier analysis demonstrated a significantly shorter time to antibody seroreversion in the experimental group, with a median time to seroreversion of 4.5 months, whereas the median was not reached in the control group. Multivariable Cox proportional hazards regression analysis indicated that Rh precision matching was independently associated with an increased likelihood of antibody seroreversion (HR = 6.12, 95% CI: 3.13–12.01, *p* < 0.001). In contrast, higher peak antibody titers were significantly associated with delayed seroreversion (Table 8).

In recurrence analyses, antibody reappearance was observed in 4 patients (8.0%) in the control group, whereas no recurrence events were recorded in the experimental group (*p* = 0.021). Patients with peak antibody titers ≥16 exhibited a significantly higher risk of recurrence (HR = 3.15, 95% CI: 1.22–8.14).

## 4 Discussion

### 4.1 The central role of the Rh system in transfusion alloimmunization

The core concept of precision transfusion lies in tailoring transfusion strategies based on an individual patient’s red blood cell antigen profile, prior alloantibody history, and transfusion exposure, with the aim of minimizing immune-mediated transfusion complications (3, 15). In international clinical practice, precision matching strategies based on the Rh five-antigen system (D, C, c, E, and e) have been increasingly applied in patient populations requiring long-term or repeated transfusions, such as pediatric oncology patients, individuals with thalassemia, and recipients of hematopoietic stem cell transplantation. These strategies have been consistently associated with a reduced incidence of alloantibody formation (16–18).

Previous studies have demonstrated that under conventional transfusion strategies limited to RhD matching, more than 30% of alloantibodies originate from non-D antigens within the Rh system (11). Our findings further support this observation. In the control group, the alloantibody formation rate was 16.7%, with anti-C and anti-E antibodies predominating, underscoring the critical role of non-D Rh antigens in transfusion-related alloimmunization.

### 4.2 Clinical and management implications of precision Rh matching

Using a prospective randomized controlled design, this study systematically evaluated the clinical performance of Rh five-antigen (D, C, c, E, e) matching in patients undergoing multiple red blood cell transfusions. Compared with conventional matching strategies, Rh five-antigen matching was significantly associated with lower rates of alloantibody formation and transfusion-related adverse reactions, suggesting a stable immunologic advantage in reducing alloimmunization risk. These findings are consistent with previous reports by Noizat-Pirenne et al. and Fasano et al., who demonstrated that prophylactic extended Rh matching (C/c, E/e) is closely associated with reduced alloimmunization in high-risk or high-responder populations (19).

A systematic review by Fasano et al. further indicated that extended Rh antigen matching is generally associated with a lower risk of alloimmunization; however, the available evidence is largely derived from observational studies with heterogeneous designs and matching strategies, highlighting the need for additional high-quality prospective data (20). In selected high-risk cohorts, comprehensive Rh (and/or Kell) matching has been reported to limit alloimmunization rates to single-digit levels or approximately 5–14%, supporting the potential clinical utility of extended matching in carefully selected populations (21).

Beyond immunologic endpoints, our study identified consistent associations between Rh five-antigen matching and several transfusion management–related outcomes. Compared with conventional matching, patients in the experimental group exhibited lower red blood cell utilization, longer transfusion intervals, and shorter hospital stays. It is important to emphasize that these parameters represent composite clinical outcomes influenced by multiple factors, including disease severity, therapeutic strategies, and clinical decision-making. Accordingly, their causal interpretation requires caution.

Further analyses demonstrated a close relationship between alloantibody formation status and transfusion efficiency. Patients who developed alloantibodies generally required higher red blood cell utilization and experienced shorter transfusion intervals. This observation is consistent with prior reports indicating that alloimmune states may reduce red blood cell survival by approximately 20–30% (22), thereby increasing transfusion requirements and management complexity. After multivariable adjustment for potential confounders, Rh five-antigen matching remained significantly associated with lower red blood cell utilization, supporting a potential management-level advantage while not implying a direct causal improvement in overall clinical prognosis.

Furthermore, the molecular genotyping results in this study provided a tool to identify potential alloimmunization risk in high-risk patients in advance. By accurately detecting partial or variant Rh antigens, transfusion teams could tailor RBC unit allocation strategies to minimize single-antigen mismatch exposure. This approach not only improves transfusion safety but also enhances the precision of managing multiply transfused patients, providing actionable guidance for clinical practice.

### 4.3 Clinical insights and biological plausibility from multivariable analyses

Multivariable logistic regression analyses identified Rh five-antigen precision matching as an independent protective factor against alloantibody formation, whereas increasing transfusion burden and the presence of underlying malignancy emerged as significant risk factors. These findings are consistent with recent studies advocating risk-stratified implementation of extended Rh matching to mitigate alloimmunization risk (1, 2). Previous work by Wemelsfelder et al. (15) and Karafin et al. (2) has similarly demonstrated that transfusion burden and underlying disease status are key independent predictors of alloantibody development.

Patients with malignancies often experience immune dysregulation due to chemotherapy or immunosuppressive treatments, rendering them more susceptible to transfusion-related alloimmune responses. Prior studies have shown that increasing transfusion frequency is strongly correlated with alloantibody risk, and that inflammatory states and impaired immune homeostasis may further amplify this risk (11). Our findings suggest that stricter precision transfusion strategies may be particularly relevant in oncology patients and those with high transfusion requirements.

From a biological perspective, the protective association of precision Rh matching may be attributed to a systematic reduction in donor–recipient antigen disparities. The Rh system exhibits substantial genetic polymorphism, and its complex genomic architecture limits the accuracy of conventional serologic phenotyping in certain contexts. Molecular genotyping enables more precise identification of Rh variants and haplotypes, thereby reducing antigen mismatches and associated immune risk (12, 16). Moreover, reduced exposure to persistent alloantigenic stimulation may attenuate prolonged T-cell helper activation, influencing the maintenance and maturation of antibody responses (3, 9, 10). It should be noted that immune signaling pathways were not directly assessed in this study, and these mechanistic interpretations are primarily based on existing literature.

### 4.4 Clinical benefits, implementation challenges, and practical feasibility

Our findings indicate that Rh five-antigen precision matching is associated with reduced alloimmunization risk and more favorable transfusion management–related outcomes, suggesting meaningful clinical value. Nevertheless, widespread implementation of this strategy must consider real-world constraints and cost implications.

At present, red blood cell molecular genotyping has not been adopted as a routine test in many institutions, and regional red blood cell antigen databases remain underdeveloped. Precision matching also imposes higher demands on blood inventory structure. Furthermore, reimbursement policies do not uniformly cover molecular genotyping costs, and awareness of non-D Rh antigen immunogenicity remains limited among some clinicians. Collectively, these factors restrict the scalability of precision transfusion strategies.

Importantly, in this study, Rh five-antigen matching was primarily achieved using established serologic methods, with molecular genotyping reserved for ambiguous or high-risk cases. This approach balanced matching accuracy with operational feasibility and cost control under existing blood bank conditions. Our findings suggest that serology-based extended Rh matching may serve as a pragmatic transitional strategy for the gradual adoption of precision transfusion.

### 4.5 Residual immunologic risk in patients receiving ≥5 transfusions

Although Rh five-antigen matching significantly reduced alloantibody formation in the overall cohort, residual immunologic risk persisted among patients receiving five or more transfusions. Trend analyses demonstrated a marked increase in alloantibody incidence with increasing transfusion frequency, highlighting a cumulative sensitization effect in heavily transfused populations (23, 24).

Previous studies have shown that, in addition to the Rh system, antigens from the Kell, Kidd, and Duffy systems also contribute substantially to alloimmunization in long-term or high-frequency transfusion recipients (16, 19, 23). Even with extended Rh matching, continued transfusion exposure may lead to alloantibody formation against non-Rh antigens (21). Accordingly, Rh five-antigen matching alone may be insufficient to fully eliminate immunologic risk in patients with extensive transfusion needs.

Based on these findings, Rh five-antigen matching may be considered a foundational protective strategy for multiply transfused patients. In high-risk subgroups, additional extension to Kell, Kidd, or Duffy antigens should be guided by individualized immunologic risk assessment and resource availability, accompanied by long-term alloantibody surveillance.

### 4.6 Strengths, limitations, and future directions

This study employed a prospective randomized controlled design with adequate sample size and relatively complete follow-up, integrating immunologic outcomes with transfusion management–related endpoints. As such, it provides comprehensive evidence supporting the application of Rh five-antigen precision matching in patients requiring multiple transfusions.

Several limitations warrant consideration. First, as a single-center study, external generalizability requires validation in multicenter and cross-regional cohorts. Second, the duration of follow-up for alloantibody outcomes was limited, and longer-term persistence and recurrence risks could not be fully characterized. Third, serologic methods were primarily used for Rh antigen determination, which may have limited sensitivity for weak or variant antigens.

Importantly, although the precision matching group demonstrated lower red blood cell utilization, longer transfusion intervals, and shorter hospital stays, these outcomes are multifactorial and subject to potential confounding. Therefore, our findings should be interpreted as demonstrating stable associations between precision Rh matching and improved transfusion management, rather than definitive causal effects.

Future studies should expand sample size through multicenter collaboration, extend follow-up duration, and systematically evaluate the long-term clinical effectiveness and cost-effectiveness of precision transfusion strategies across diverse high-risk populations. The development of regional or national red blood cell antigen databases integrated with transfusion information systems may further enhance the feasibility and clinical impact of precision transfusion.

Overall, Rh five-antigen precision matching appears most appropriate as a targeted strategy for patients requiring repeated or high-risk transfusions, rather than as a universal approach for all transfusion recipients.

## 5 Conclusion

In patients requiring multiple red blood cell transfusions, Rh five-antigen precision matching was significantly associated with a reduced risk of alloantibody formation and improvements in several transfusion management–related outcomes.

This strategy represents a feasible transfusion management option for patients at high immunologic risk. Its long-term clinical value and cost-effectiveness across different patient populations warrant further investigation.

## Data Availability

The raw data supporting the conclusions of this article will be made available by the authors, without undue reservation.
